# A conversation with Dr. Lesley A. Inker, MD, MS, Director, Kidney and Blood Pressure Center; Associate Professor, Tufts Medical Center; Tufts University School of Medicine

**DOI:** 10.1017/cts.2022.522

**Published:** 2023-02-15

**Authors:** Kathy Siranosian

**Affiliations:** Clinical Research Forum, Washington, DC, USA

## Top 10 Clinical Research Achievement Awards Q & A

This article is part of a series of interviews with recipients of Clinical Research Forum’s Top 10 Clinical Research Achievement Awards. This article is with Dr. Lesley A. Inker, MD, MS, Director, Kidney and Blood Pressure Center; Associate Professor, Tufts Medical Center; Tufts University School of Medicine. Dr. Inker studies the measurement and estimation of kidney function, epidemiology of chronic kidney disease (CKD), biomarkers for CKD progression, and treatments to slow the progression of CKD. Dr. Inker received a 2022 Top 10 Clinical Research Achievement Award for *New Creatinine- and Cystatin C-Based Equations to Estimate GFR without Race* [1]. *The interview has been edited for length and clarity.*

## When Did You First Become Interested in Clinical Research?

My father was a physician, so I grew up knowing about medicine, but I didn’t get experience with research until I was an undergraduate at McGill University. I majored in psychology and did an honors thesis with a professor who was studying language acquisition by comparing sign versus spoken languages. She was a fantastic role model. Working in her lab helped me discover that I loved research, and the process of scientific inquiry. From there, I went on to medical school, but with the full intention to be a physician investigator. I started out initially interested in specializing in neurology because of the experience as an undergraduate, and then learned I liked the complexity of the human body as captured in internal medicine, which eventually led me to nephrology. All along, my primary focus has been preventing disease. Now, as a clinical researcher and a nephrologist, I’m working every day to prevent the progression of chronic kidney disease (CKD).

## Your Award-Winning Study Is about the Development of New Equations for Estimated Glomerular Filtration Rate (eGFR) Without Race. What Are the Challenges Associated with Evaluating the Accuracy of Current Guideline-Recommended Equations?

Our goal is always to use the data we have to make the most informed decisions that lead to the best patient outcomes. We have to stay open to change. Incorporation of new data is important as our society changes. This can be challenging – studies require funding, they take a long time to complete, and it can be difficult to collect data and samples from diverse populations. Even so, it’s what we need to do to make sure our standards best reflect society. How we think about disease prevention and management must be continually evolving.

## Are We Getting Better at That?

Yes, but there will always be challenges. For instance, my hope is that electronic medical records will help. They provide enormous amounts of data and I’m part of a consortium that’s looking at adverse events related to medications focusing primarily on CKD. We're able to use data from patients around the world, which we would not have been able to do a decade ago. However, the data in electronic medical records can only answer selected questions. To measure GFR, patients have to come in for a study visit and timed samples have to be taken. These studies require planning and funding. We’re thinking now about new more accurate ways to estimate kidney function that just require blood samples. Ultimately, this would make it easier to conduct studies and get samples from diverse populations, but these new methods must first be validated against the measured GFR.

## Is This Where Kidney Disease Research Is Heading?

We are working on several studies aimed at finding new markers for kidney function. These studies involve global metabolomics, and our goal is to find novel metabolites that can estimate kidney function more accurately. The field of metabolomics can also give us insight into disease progression and possible treatments. Of course, estimating kidney function is just one aspect of researching kidney disease. For example, we know a lot more about the genetics of CKD than we did even 5 years ago, which may also help us to develop disease-specific treatments

## Please Tell Us about the Collaborations that Support Your Research

Our group is called the Chronic Kidney Disease Epidemiology Collaboration. When I talk about our work, I always say that the strength of our data speaks to the strength of the collaborative nature of our epidemiology community. I’m immensely grateful and very proud of this collaboration, and we’re just one of many in nephrology. I think we all understand that we can only do big work together. The research also requires partnerships with clinical chemistry, biostatisticians, and other clinicians, and we’ve had wonderful, engaged partners over the years. I’ve learned so much from them and they’ve been critical to our success. It’s the collaborations that make our work as impactful as it has been – and a lot of fun, too.

## What Advice Can You Share for Someone Who Is Just Starting Their Career or Thinking about Starting Their Career as a Clinical Researcher?

I got great advice when I was starting: Find someone who is asking the right questions and work with them. When you’re asking the right questions, your work has the potential to have the biggest impact on patients, and that’s incredibly motivating. Beyond that, find people you really like to work with. Being a clinical researcher is very rewarding, but it does not lead to instant gratification. Success can be a long haul, but when you’re working with people you like, the process itself is worthwhile.
